# Supplementary material for: FANCD2 modulates the mitochondrial stress response to prevent common fragile site instability
Source: Commun Biol. 2021 Jan 29;4:127. doi: 10.1038/s42003-021-01647-8 (PMC7846573; doi:10.1038/s42003-021-01647-8)
Supplement: Supplementary file 2 — Supplementary Information [file 42003_2021_1647_MOESM2_ESM.pdf]

Supplementary Fig. 1

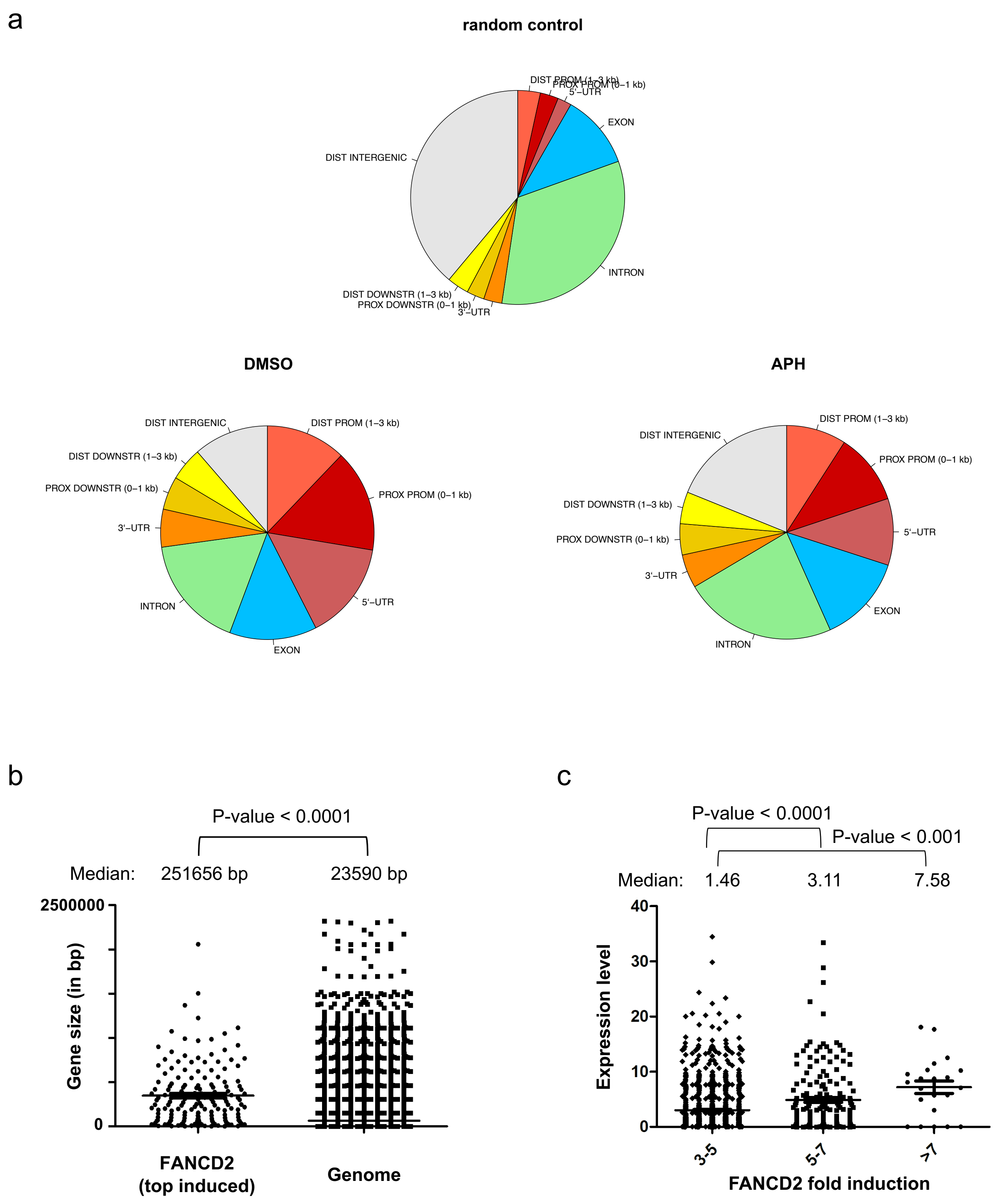

Supplementary Fig. 1: FANCD2 binds to gene regions in a manner correlated with transcription.

a) FANCD2 location peaks relative to genomic annotations are represented in a pie chart. As a control, randomly located “peaks” were also run against the same genomic features database.

b) Comparison of the median gene size between genes most enriched in FANCD2 after APH treatment (top-induced) and all genes present in the genome.

c) FANCD2 binding fold induction according to the gene expression level. Gene expression data were obtained from ENCODE: Experimental Summary ENCSR000CWM. Gene expression values are in log2 FPKM (Fragments Per Kilobase of exon per Million reads) units.

Supplementary Fig. 2

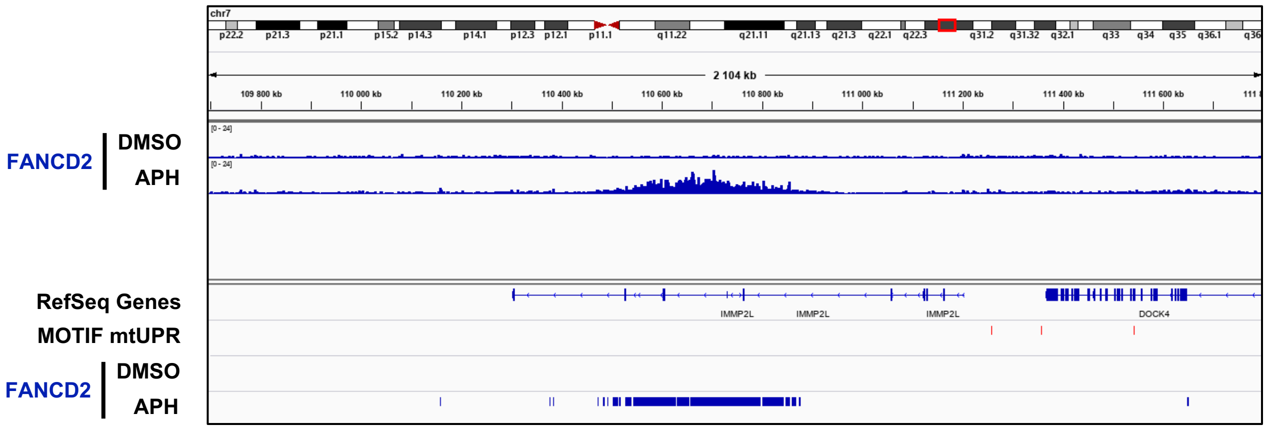

Supplementary Fig. 2: FANCD2 location relative to mtUPR motifs.

IGV visualization of FANCD2 enrichment along the CFS gene *IMMP2L* in the presence or absence of APH and location of mitochondrial UPR (mtUPR) motifs (red tags) and FANCD2 peaks.

Supplementary Fig. 3

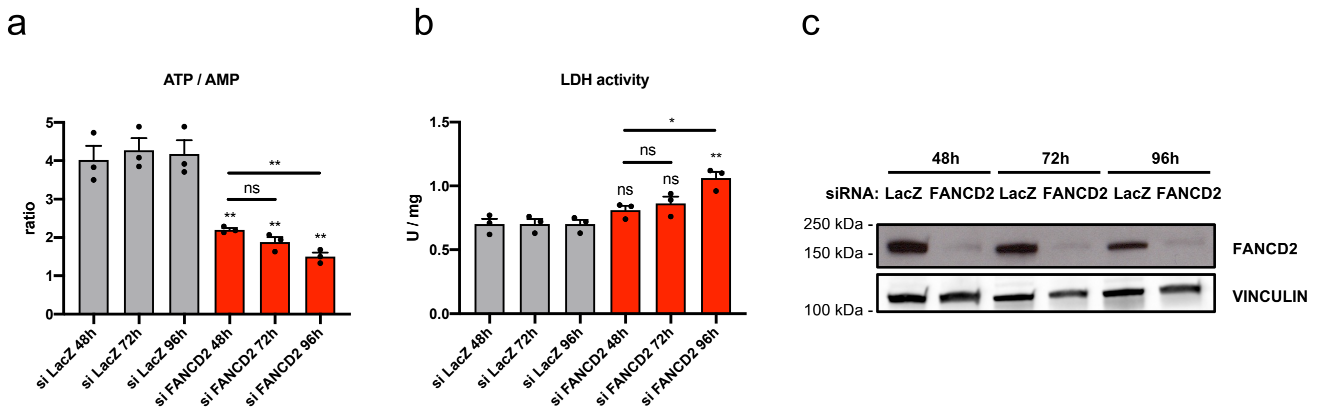

Supplementary Fig. 3: FANCD2 depletion induces a metabolic switch.
ATP/AMP ratio (a) and LDH activity (b) measured at 48, 72 and 96 hr after transfection with control or FANCD2 siRNA. c) Western blot of whole-cell lysate of control and FANCD2 siRNA-transfected cells at the indicated time points after transfection (right). n=3 independent experiments. a) **p=0.008 (siFANCD2_48h), **p=0.0022 (siFANCD2_72h), **p=0.0021 (siFANCD2_96h), **p=0.0034 (siFANCD2_96h vs siFANCD2_48h). b) **p=0.0044 (siFANCD2_96h), *p=0.0152 (siFANCD2_96h vs siFANCD2_48h). Error bars are SEM.

Supplementary Fig. 4

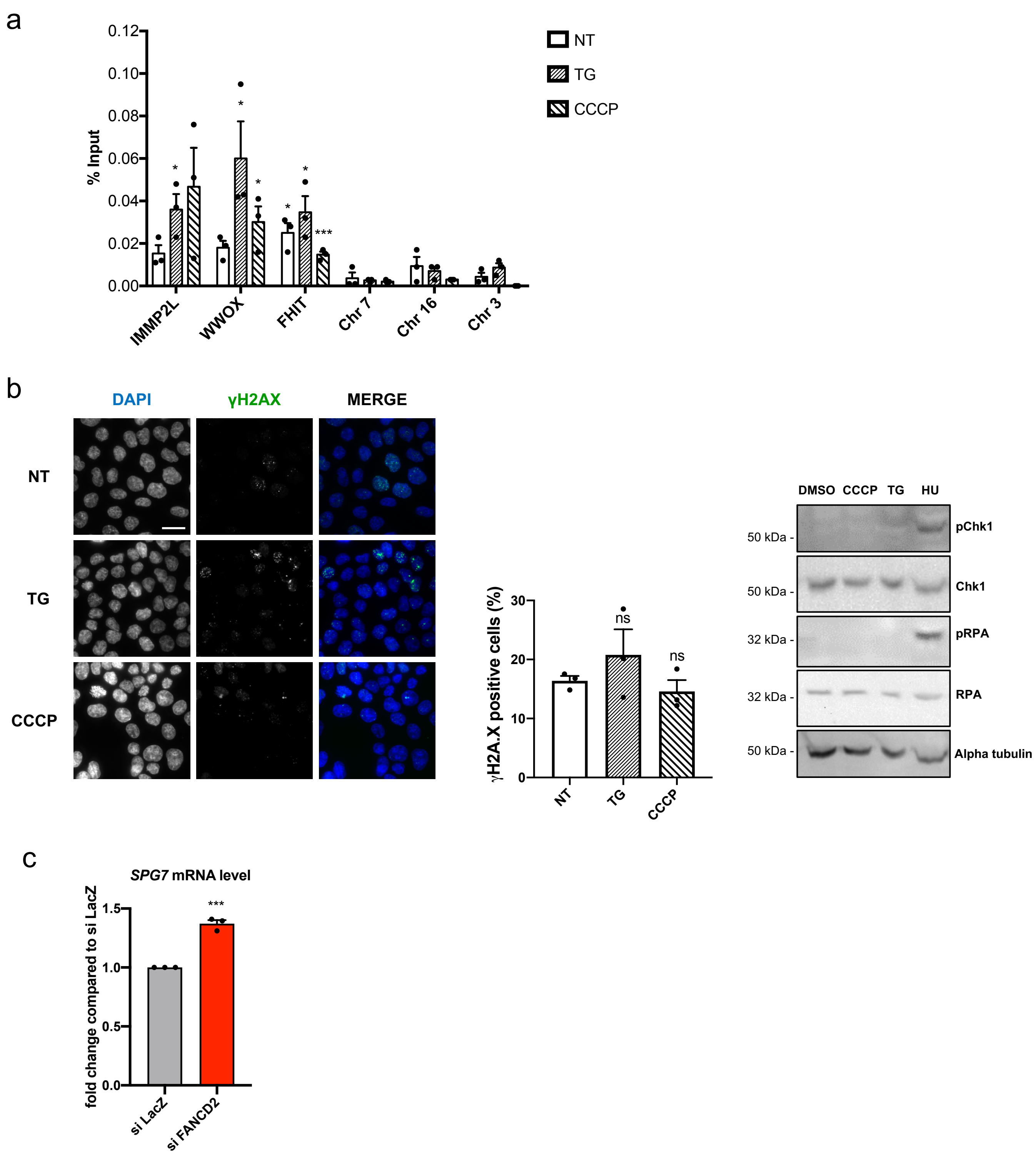

Supplementary Fig. 4: Recruitment of FANC protein after mitochondrial or ER stress in absence of obvious DNA damage response.

a) FANCI ChIP followed by qPCR in cells treated or not with 1 μM TG or 10 μM CCCP for 8 hr. The results are expressed as the percentage of the input. Chromosome 7 (Chr 7), Chromosome 16 (Chr 16), and Chromosome 3 (Chr 3) were used as control regions close to the *IMMP2L*, *WWOX*, and *FHIT* genes, respectively. n=3 independent experiments. *p=0.01 (IMMP2L_TG), *p=0.0396 (WWOX_TG), *p=0.0215 (WWOX_CCCP), *p=0.0139 (FHIT_NT), *p=0.03 (FHIT_TG), ***p=0.0005 (FHIT_CCCP).

b) Immunofluorescence analysis of γH2AX nuclear foci in cells treated or not (NT) with 1 μM TG or 10 μM CCCP for 8 hr (left panel). Scale bar, 20 μM. Quantification of the percentage of γH2AX-positive cells in each condition is reported in middle panel. Right panel: immunoblot detection of phospho-S33 RPA2 (pRPA) and phospho-S345 chk1 (pChk1) in control (DMSO), TG or CCCP-treated cells. Hydroxyurea (HU) treatment (2mM, 30h) was used to control for checkpoint activation.

c) mRNA levels of SPG7 measured by RT-qPCR after siFANCD2 transfection, compared to levels after siLacZ transfection. ***p=0.003. Error bars are SEM.

Supplementary Fig. 5

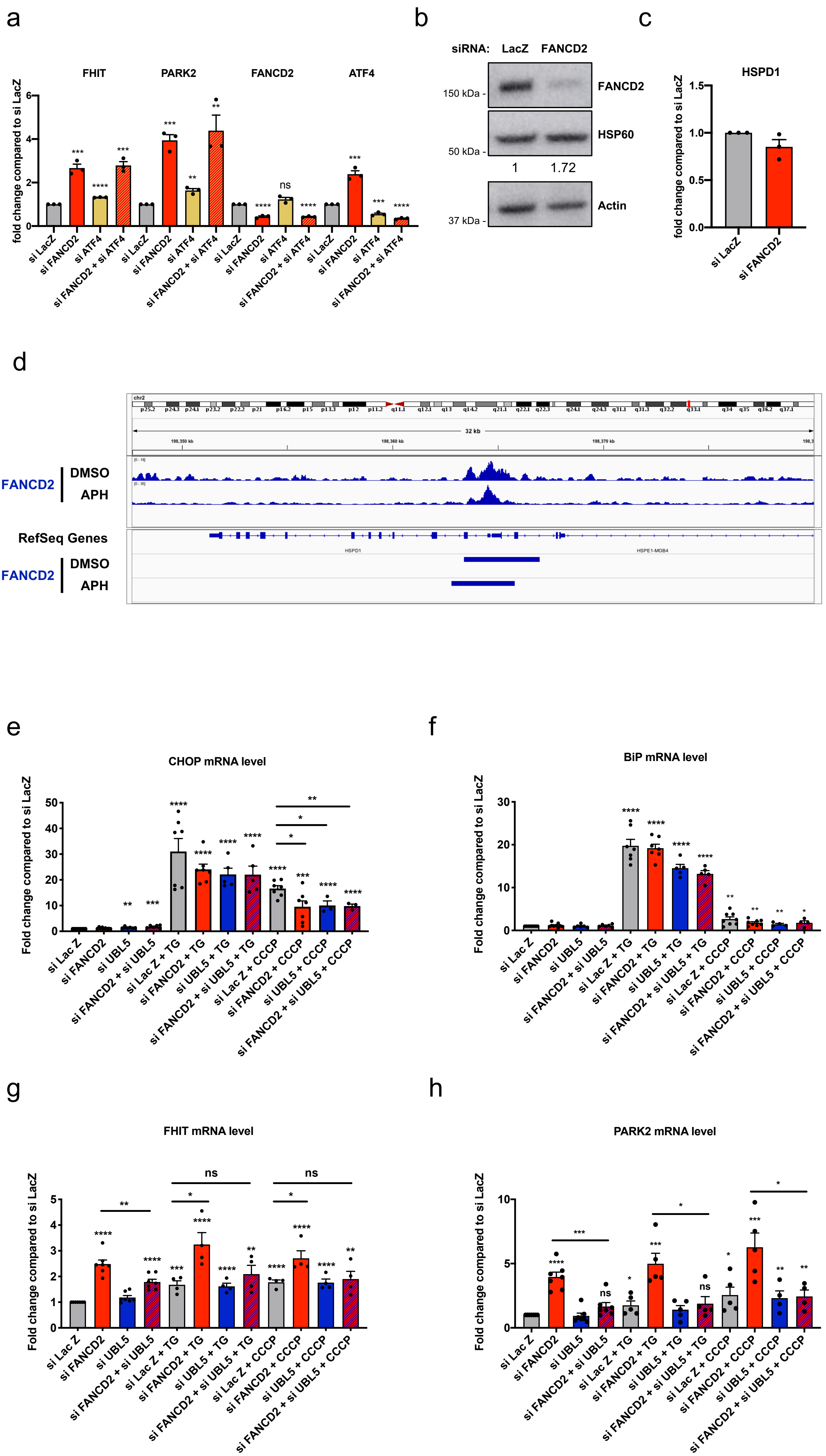

Supplementary Fig. 5: ATF4 and FANCD2-UBL5 regulate distinct branches of the mitochondrial stress response.

a) Analysis of CFS gene expression measured by RT-qPCR after control (siLacZ), FANCD2, ATF4, or FANCD2 and ATF4 siRNA transfection. The relative mRNA levels of *FANCD2* and *ATF4* were verified in the same experiments. n= 3 independent experiments.

b) Western blot of whole-cell lysate of control and FANCD2 siRNA-transfected cells showing the HSP60 protein levels in each condition. Quantification of the HSP60 relative abundance normalized to the loading control (actin) is reported under the corresponding band.

c) HSPD1 mRNA level measured by RT-qPCR after control (siLacZ) or FANCD2 siRNA transfection. n=3 independent experiments.

d) IGV visualization of FANCD2 enrichment at the common bidirectional promoter of *HSPD1 and HSPE1* genes, encoding the mitochondrial chaperonins HSP60 and HSP10, respectively.

e) CHOP mRNA level measured by RT-qPCR in siLacZ-, siFANCD2-, siUBL5- or siFANCD2 and siUBL5 transfected cells treated or not with 1 µM TG or 10 µM CCCP for 8 hr. n=9 (siLacZ, siFANCD2), n=7 (siLacZ + TG, siFANCD2 + TG, siLacZ + CCCP, siFANCD2 + CCCP), n=6 (siUBL5, siFANCD2 + UBL5), n=5 (siUBL5 + TG, siFANCD2 + siUBL5 + TG), n=3 (siUBL5 + CCCP, siFANCD2 + siUBL5 + CCCP) independent experiments.

f) BiP mRNA level measured by RT-qPCR in siLacZ-, siFANCD2-, siUBL5- or siFANCD2 and siUBL5 transfected cells treated or not with 1 µM TG or 10 µM CCCP for 8 hr. n=9 (siLacZ, siFANCD2), n=8 (siLacZ + CCCP, siFANCD2 + CCCP), n=7 (siLacZ + TG, siFANCD2 + TG), n=6 (siUBL5, siFANCD2 + UBL5), n=5 (siUBL5 + TG, siFANCD2 + siUBL5 + TG), n=4 (siUBL5 + CCCP, siFANCD2 + siUBL5 + CCCP) independent experiments.

g) FHIT mRNA level measured by RT-qPCR in siLacZ-, siFANCD2-, siUBL5- or siFANCD2 and siUBL5 transfected cells treated or not with 1 µM TG or 10 µM CCCP for 8 hr. n=7 (siLacZ, siFANCD2, siUBL5, siFANCD2 + UBL5), n=4 (siLacZ + TG, siFANCD2 + TG, siUBL5 + TG, siFANCD2 + siUBL5 + TG, siLacZ + CCCP, siFANCD2 + CCCP, siUBL5 + CCCP, siFANCD2 + siUBL5 + CCCP) independent experiments.

h) PARK2 mRNA level measured by RT-qPCR in siLacZ-, siFANCD2-, siUBL5- or siFANCD2 and siUBL5 transfected cells treated or not with 1 µM TG or 10 µM CCCP for 8 hr. n=7 (siLacZ, siFANCD2, siUBL5, siFANCD2 + UBL5), n=5 (siLacZ + TG, siFANCD2 + TG, siUBL5 + TG, siFANCD2 + siUBL5 + TG, siLacZ + CCCP, siFANCD2 + CCCP), n=4 (siUBL5 + CCCP, siFANCD2 + siUBL5 + CCCP) independent experiments. Error bars are SEM.

Supplementary Figure 6: unprocessed blots

Figure 1d

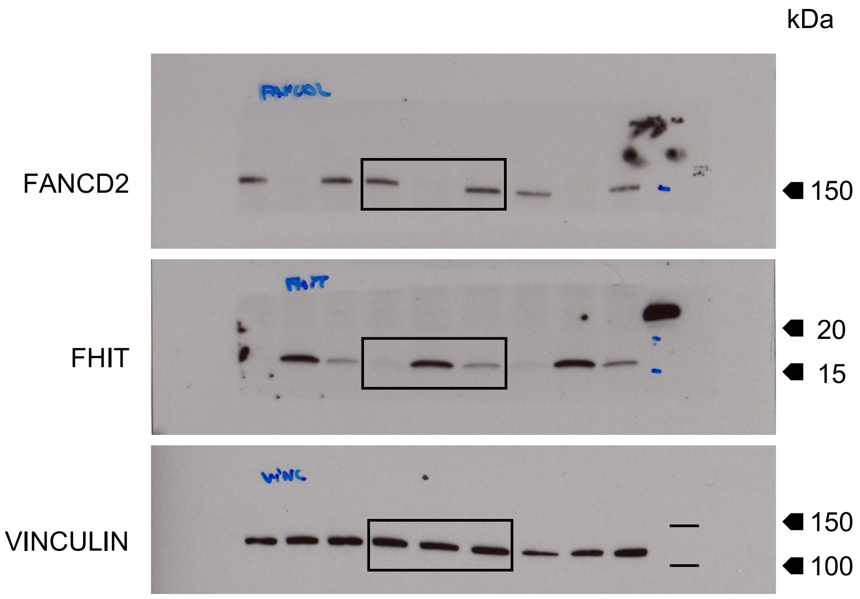

Figure 1f

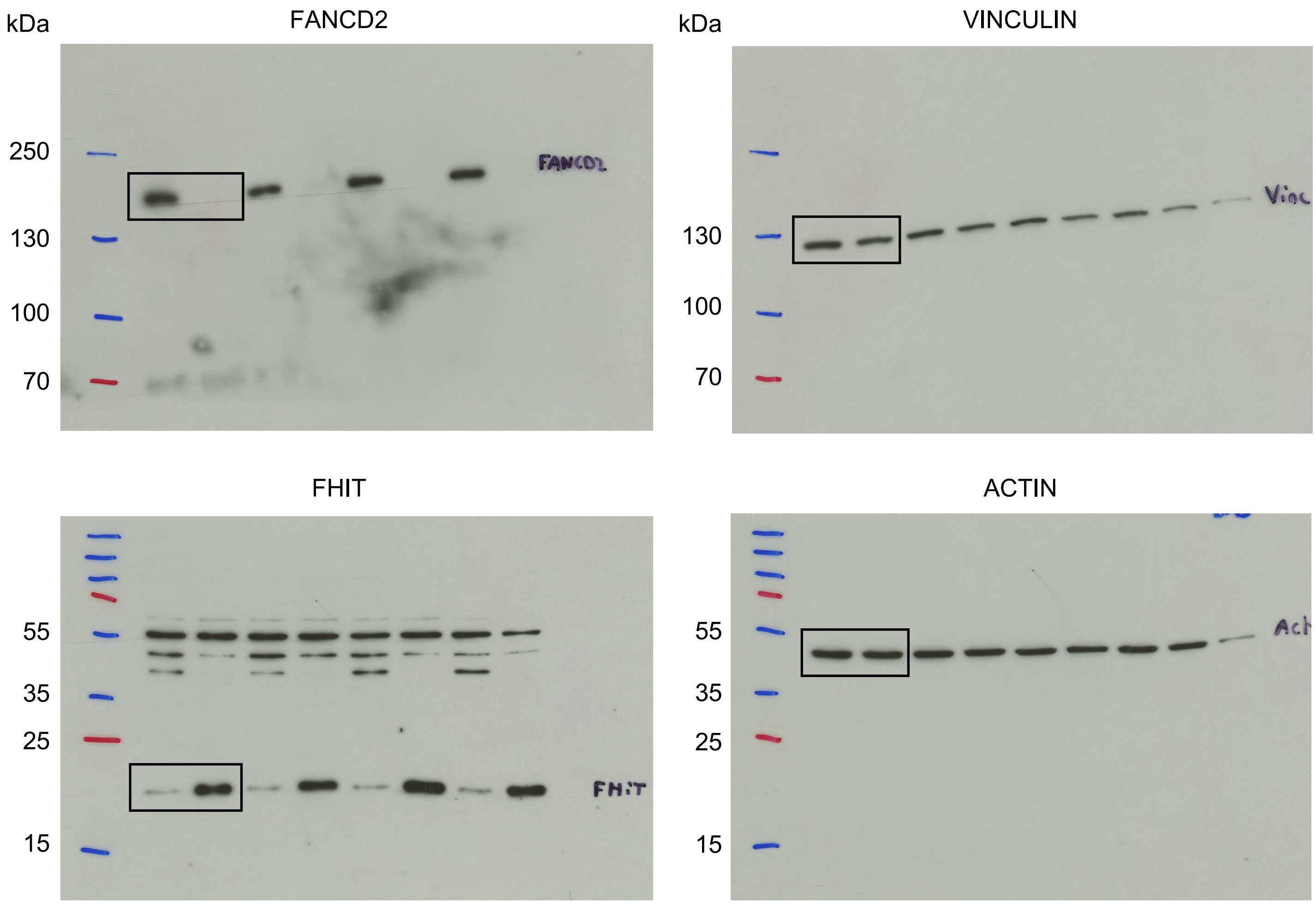

Figure 1g

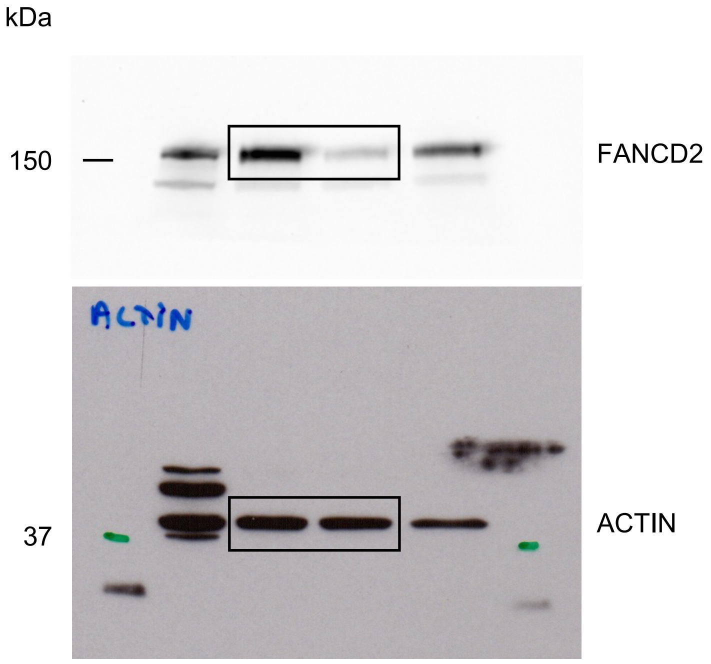

Figure 1h

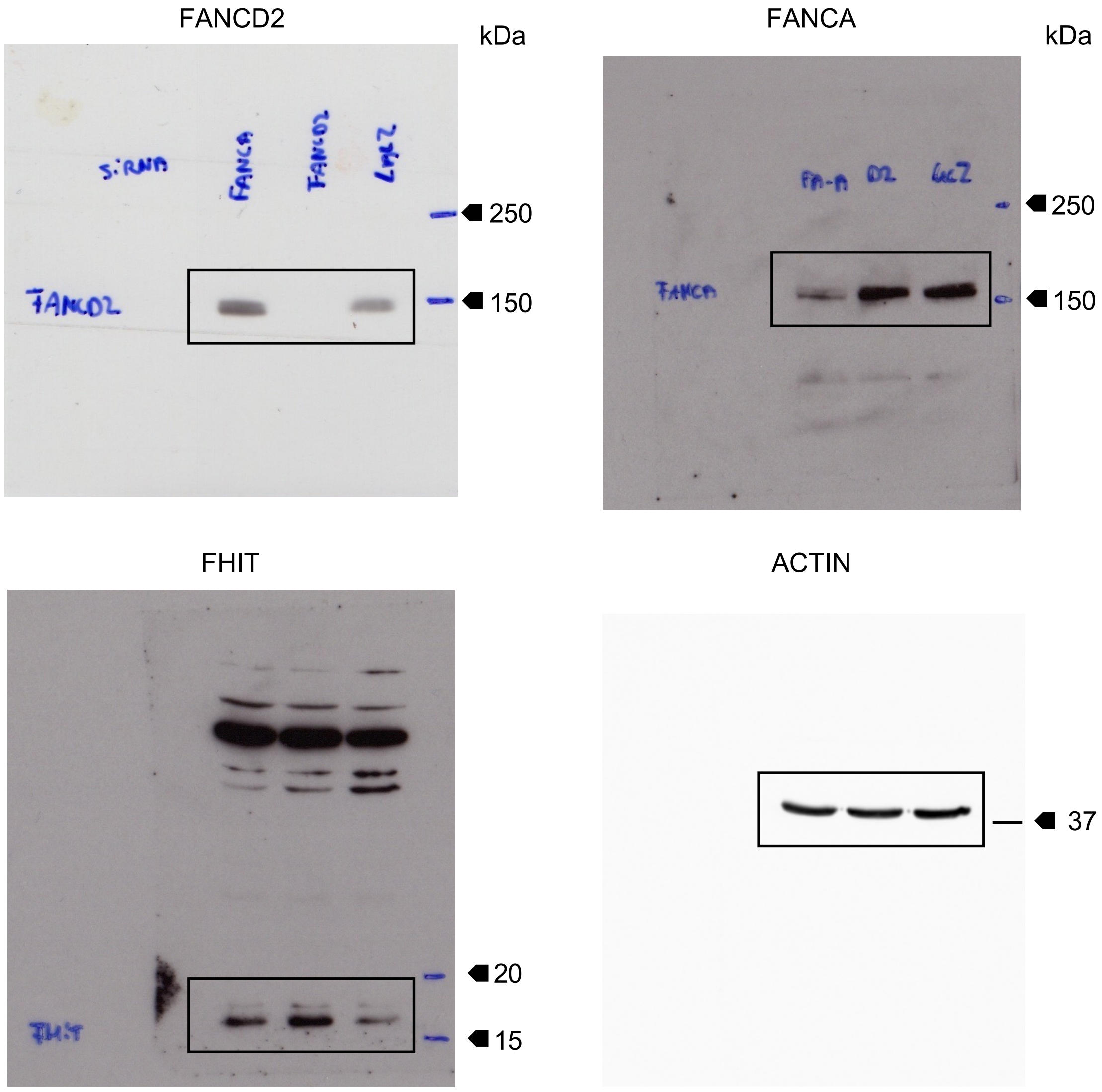

Figure 1i

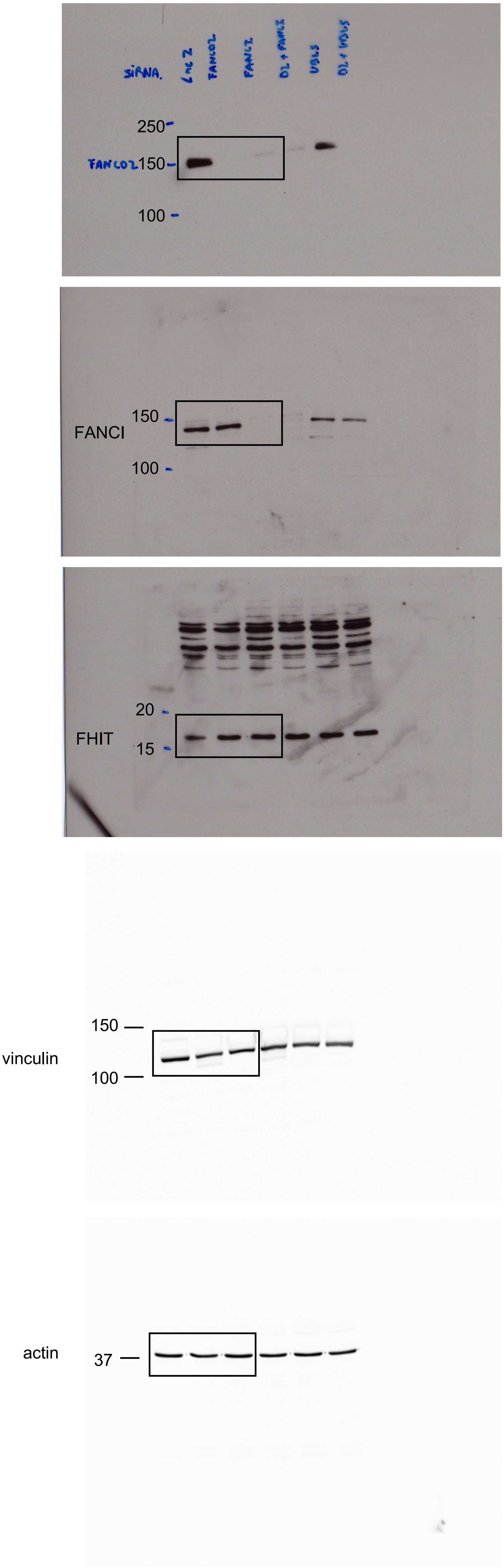

siRNA.
250
FANCD2
150
100
150
FANCI
100
20
FHIT
15
150
vinculin
100
actin
37

Fig. 5g

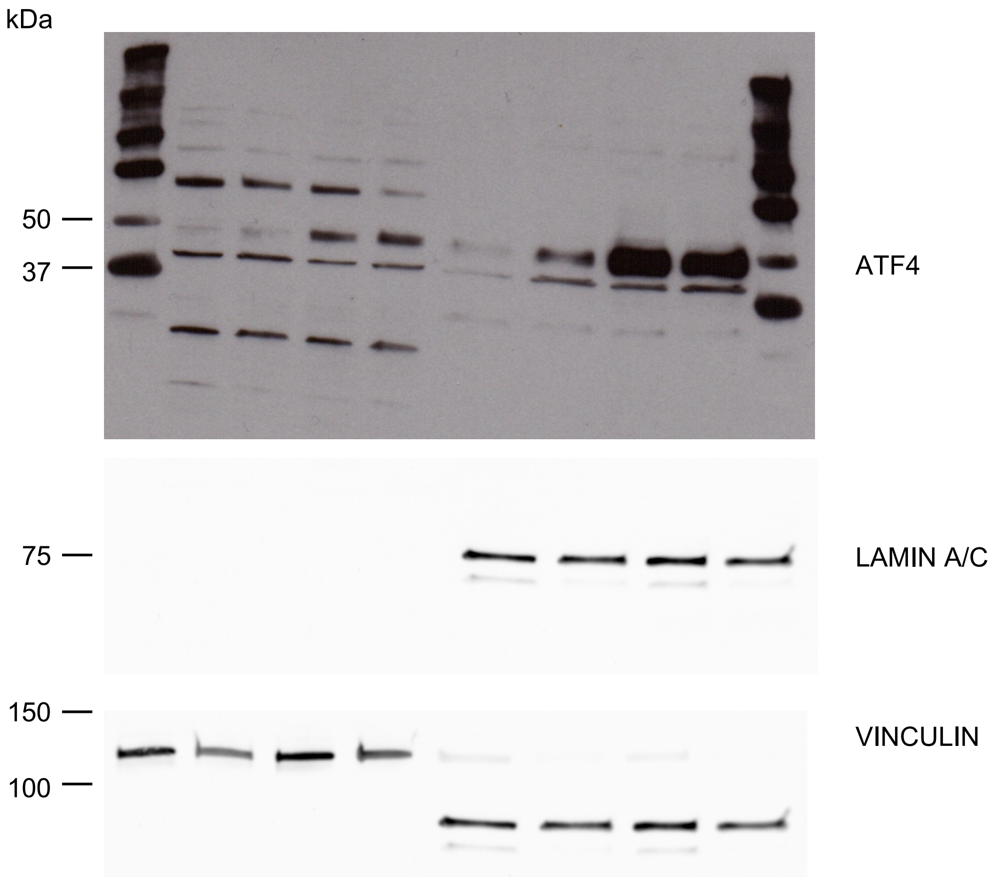

Supplementary Fig. 3

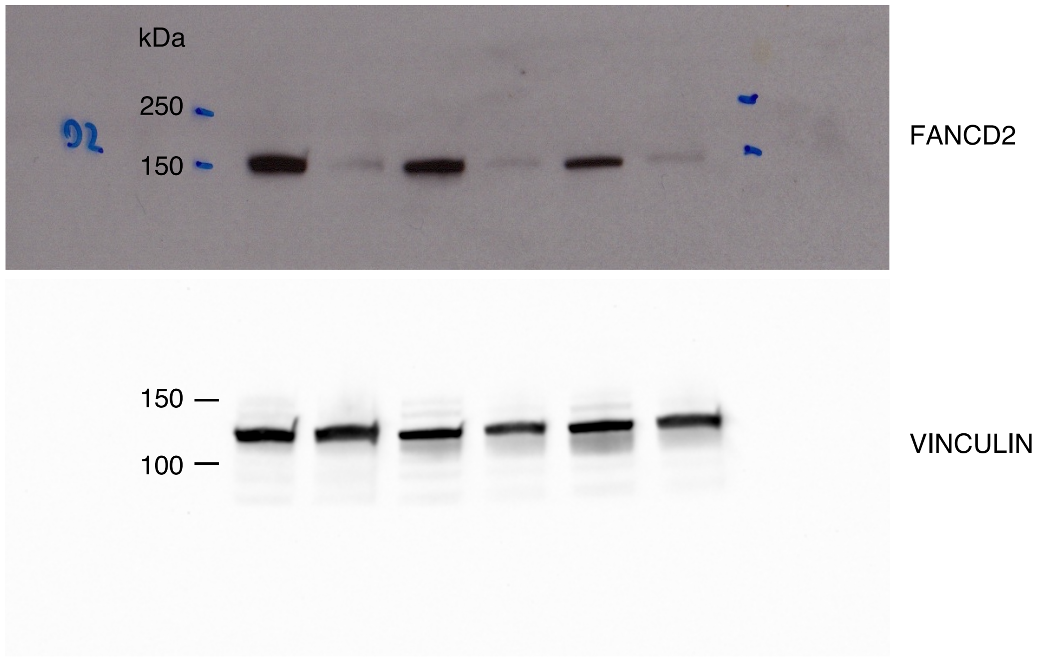

Supplementary Fig. 4b

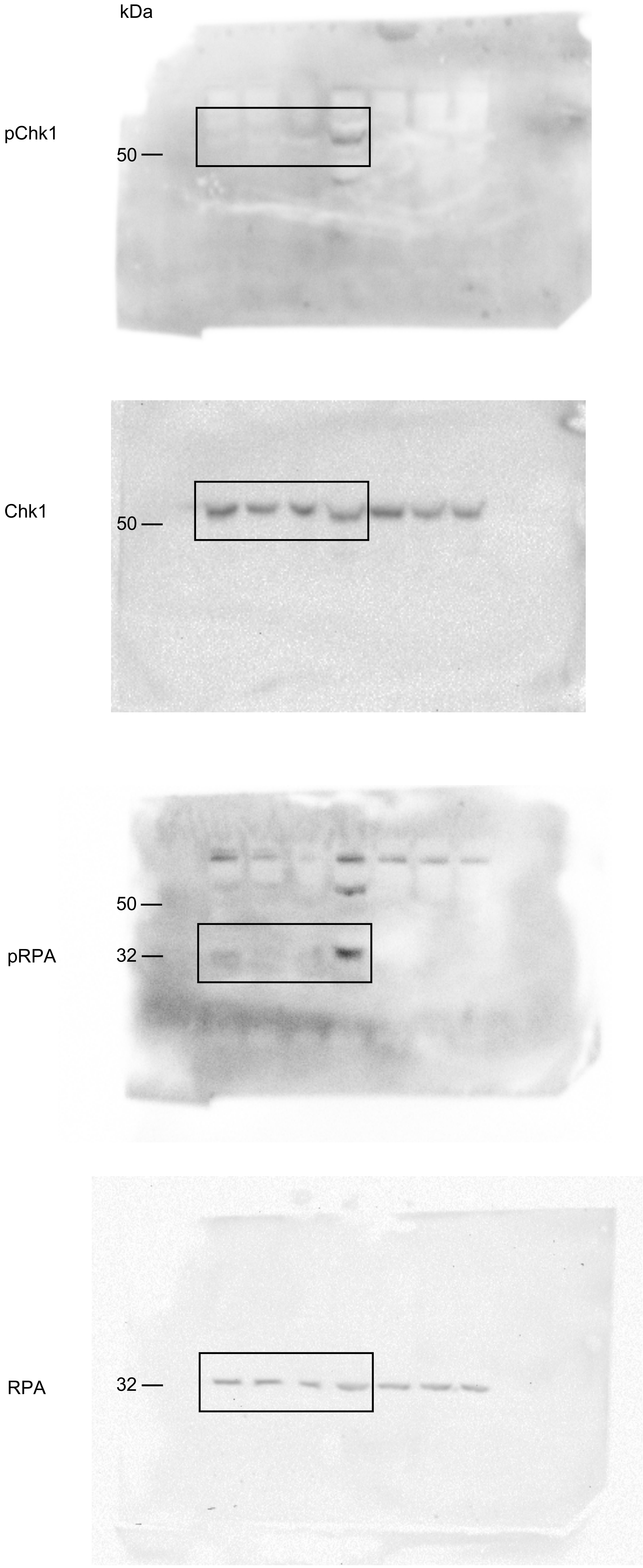

Supplementary Fig. 4b

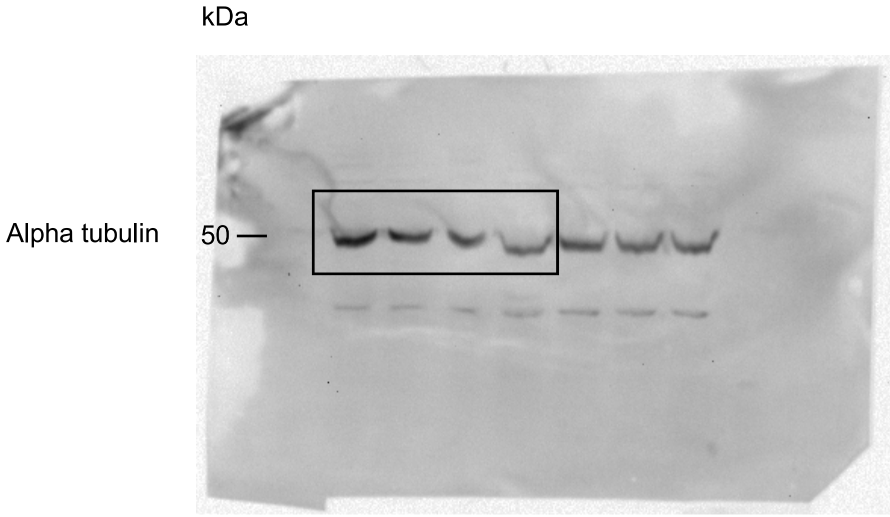

Supplementary Fig. 5b

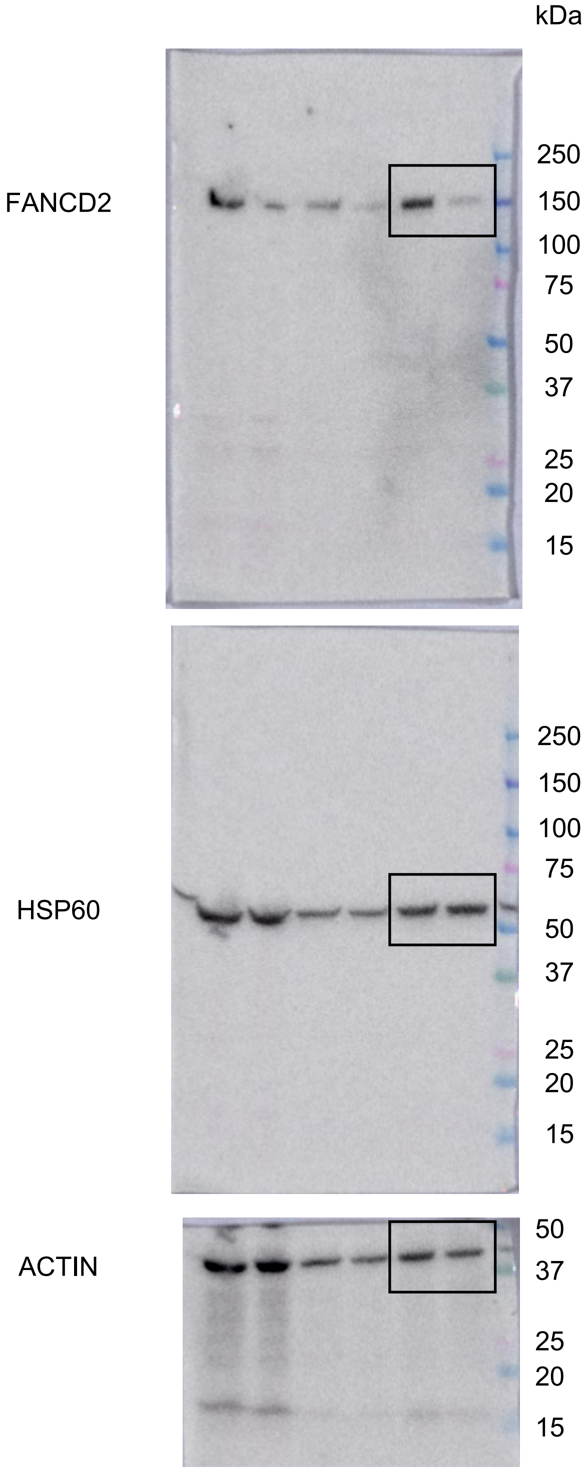

Supplementary Table S1

siRNA sequences

siRNA	Sequence		
LacZ	CGU-CGA-CGG-AAU-ACU-UCG-A		
FANCD2	GGA-GAU-UGA-UGG-UCU-ACU-A		
FANCD2 #2	GAU-AAG-UUG-UCG-UCU-AUU-A		
FANCA (mix of 4 sequences)	GUU-AGA-GUU-UGC-UCA-GUA-U		
	GAG-CCG-UGC-AGA-UCU-GUC-C		
	CGC-UUU-GGC-UGC-UGG-AGU-A		
	CGA-CAU-GCA-UGC-UGU-GGG-A		
ATF4	CCA-CGU-UGG-AUG-ACA-CUU-G		
UBL5	CCU-GGA-GCU-UUA-UUA-UCA-A		
SPG7	CCU-CAA-GGU-UGA-AGC-AGA-AGA-AUA-A		

Antibodies

Protein	Company	Reference
ACTIN	Santa Cruz	1616
ATF4	Proteintech	10835-1-AP
FANCA	Abcam	AB97578
FANCD2 (ChIP)	Novus Biologicals	NB100-182
FANCD2 (IF)	Abcam	AB108928
FANCD2 (WB)	Santa Cruz	20022
FANCI	Novus Biologicals	NB100-60447
FHIT	Thermo Fisher	71-9000
LAMIN A/C	Santa Cruz	7292
VINCULIN	Abcam	AB18058
Chk1 phospho-S345	Cell Signaling	2348
RPA2 phospho-S33	Bethyl	A300-246A-M
RPA (34-19)	Calbiochem	NA18
Chk1	Cell Signaling	2360
Alpha-Tubulin	SIGMA	T6199
HSP60	Enzo	ADI-SPA-806

q-PCR primer sequences

	Forward	Reverse
**mRNA expression**		
ATF4	GTT-CTC-CAG-CGA-CAA-GGC-TA	ATC-CTG-CTT-GCT-GTT-GTT-GG
CHAC1	GTG-GTG-ACG-CTC-CTT-GAA-GA	TTC-AGG-GCC-TTG-CTT-ACC-TG
FANCD2	CCA-TGG-TCA-CAG-CAC-CAA-TA	TCA-GCA-CAC-TGG-CAT-TTA-GC
FHIT	CGT-TCA-CGT-CCA-TGT-TCT-TC	CTC-CAA-GAG-GCA-GGA-AAG-TC
GAPDH	CCT-CAA-CGA-CCA-CTT-TGT-CA	TTC-CTC-TTG-TGC-TCT-TGC-TG
IMMP2L	TGA-AGG-AGA-TAT-TGT-CAG-AAC-CAT-AGG	GAT-CAC-CTT-CAA-CCC-AGA-TGT-GA
PARK2	ACC-TCA-GCA-GCT-CAG-TCC-TC	TGC-TGC-ACT-GTA-CCC-TGA-GT
PCK2	CAT-CCG-AAA-GCT-CCC-CAA-GT	GCT-CTC-TAC-TCG-TGC-CAC-AT
PSAT1	GTC-CAG-TGG-AGC-CCC-AAA-A	TGC-CTC-CCA-CAG-ACC-TAT-GC
U1	CCC-AGG-GCG-AGG-CTT-ATC-CA	CGA-ACG-CAG-TCC-CCC-ACT-AC
WWOX	AGT-GGC-AGG-AGA-TTT-GCC-AT	GTG-ACC-ACA-ACC-ACT-TTG-GC
SPG7	AAG-GGA-TCA-ACG-GAT-TGT-TGT-T	AGC-CGC-TCT-CGG-TAC-ATC-T
CHOP	AGA-ACC-AGG-AAA-CGG-AAA-CAG-A	TCT-CCT-TCA-TGC-GCT-GCT-TT
BiP	TGT-TCA-ACC-AAT-TAT-CAG-CAA-ACT-C	TTC-TGC-TGT-ATC-CTC-TTC-ACC-AGT
UBL5	GGA-TGA-TCG-AGG-TTG-TTT-GCA	CTT-CTT-AAG-GTC-CCC-GAT-GGT
HSPD1	CTT-TTA-GCC-GAT-GCT-GTG-GC	GGA-CTT-CCC-CAA-CTC-TGC-TC
**ChIP primers**		
IMMP2L	TCC-TCC-TGG-CTG-CTT-TCA-TG	GAG-CTG-TGA-GAA-GTG-TGC-CA
WWOX	CCA-CCT-TTT-GCC-GCA-GTA-AC	AGG-CCC-TGA-TGA-CAT-CTC-CT
FHIT	AGG-CAA-GTT-GAC-AAC-ACC-CA	CGA-AGC-CAG-GGT-TTG-CAA-TC
Chr7	ACC-TGA-CAG-AAC-CCA-GAT-GC	TGT-GGG-AGT-GAG-GGA-AAA-AG
Chr16	AAC-CAC-CCT-CCA-CAA-GAC-TG	CCG-TCT-CAA-TAG-GAG-GGA-CA
Chr3	CGC-ACT-TGC-TAT-TCC-CTC-AT	AAG-CTG-GCC-TGT-GAG-TAG-GA
