# Supplementary material for: FANCD2 modulates the mitochondrial stress response to prevent common fragile site instability
Source: Commun Biol. 2021 Jan 29;4:127. doi: 10.1038/s42003-021-01647-8 (PMC7846573; doi:10.1038/s42003-021-01647-8)
Supplement: Supplementary file 3 — Description of Additional Supplementary Files [file 42003_2021_1647_MOESM3_ESM.pdf]

Description of Additional Supplementary Files

File name: Supplementary Data 1

Description: Source data underlying the graphs presented in the article Figures as Supplementary Data 1.
