# Supplementary material for: FANCD2 modulates the mitochondrial stress response to prevent common fragile site instability
Source: Commun Biol. 2021 Jan 29;4:127. doi: 10.1038/s42003-021-01647-8 (PMC7846573; doi:10.1038/s42003-021-01647-8)
Supplement: Supplementary file 5 — Reporting Summary [file 42003_2021_1647_MOESM5_ESM.pdf]

nature research

Corresponding author(s): Valeria Naim

Last updated by author(s): Dec 12, 2020

Reporting Summary

Nature Research wishes to improve the reproducibility of the work that we publish. This form provides structure for consistency and transparency in reporting. For further information on Nature Research policies, seeAuthors & Referees and theEditorial Policy Checklist .

Statistics

For all statistical analyses, confirm that the following items are present in the figure legend, table legend, main text, or Methods section.

n/a	Confirmed	
☐	☒	The exact sample size (*n*) for each experimental group/condition, given as a discrete number and unit of measurement
☐	☒	A statement on whether measurements were taken from distinct samples or whether the same sample was measured repeatedly
☐	☒	The statistical test(s) used AND whether they are one- or two-sided<br>*Only common tests should be described solely by name; describe more complex techniques in the Methods section.*
☒	☐	A description of all covariates tested
☒	☐	A description of any assumptions or corrections, such as tests of normality and adjustment for multiple comparisons
☐	☒	A full description of the statistical parameters including central tendency (e.g. means) or other basic estimates (e.g. regression coefficient) AND variation (e.g. standard deviation) or associated estimates of uncertainty (e.g. confidence intervals)
☐	☒	For null hypothesis testing, the test statistic (e.g. *F*, *t*, *r*) with confidence intervals, effect sizes, degrees of freedom and *P* value noted<br>*Give P values as exact values whenever suitable.*
☒	☐	For Bayesian analysis, information on the choice of priors and Markov chain Monte Carlo settings
☒	☐	For hierarchical and complex designs, identification of the appropriate level for tests and full reporting of outcomes
☒	☐	Estimates of effect sizes (e.g. Cohen's *d*, Pearson's *r*), indicating how they were calculated

Our web collection on statistics for biologists contains articles on many of the points above.

Software and code

Policy information about availability of computer code

Data collection	No software was used.
Data analysis	For ChIP-seq data, peaks were called using the SICER algorithm and aligned to the human genome build hg19. Integrative Genomics Viewer (IGV) was used to visualize peaks from the genome. FANCD2 ChIP seq data were further scanned with RSAT (Regulatory Sequence Analysis Tools)-matrix-scan to identify regulatory motifs.

For manuscripts utilizing custom algorithms or software that are central to the research but not yet described in published literature, software must be made available to editors/reviewers. We strongly encourage code deposition in a community repository (e.g. GitHub). See the Nature Research guidelines for submitting code & software for further information.

Data

Policy information about availability of data

All manuscripts must include a data availability statement. This statement should provide the following information, where applicable:

- Accession codes, unique identifiers, or web links for publicly available datasets
- A list of figures that have associated raw data
- A description of any restrictions on data availability

ChIP-seq data have been deposited on GEO: GEO accession GSE141101 in private status and a secure token is available.

Field-specific reporting

Please select the one below that is the best fit for your research. If you are not sure, read the appropriate sections before making your selection.

Life sciences study design

All studies must disclose on these points even when the disclosure is negative.

Sample size	No statistical methods were used to determine sample size.
Data exclusions	No data was excluded from the analysis.
Replication	All data are the results of at least three independent experiments and n is indicated in the Figure legends.
Randomization	Not applicable
Blinding	Investigators were not blinded.

Reporting for specific materials, systems and methods

We require information from authors about some types of materials, experimental systems and methods used in many studies. Here, indicate whether each material, system or method listed is relevant to your study. If you are not sure if a list item applies to your research, read the appropriate section before selecting a response.

Materials & experimental systems

n/a	Involved in the study	
☐	☒	Antibodies
☐	☒	Eukaryotic cell lines
☒	☐	Palaeontology
☒	☐	Animals and other organisms
☒	☐	Human research participants
☒	☐	Clinical data

Methods

n/a	Involved in the study	
☐	☒	ChIP-seq
☒	☐	Flow cytometry
☒	☐	MRI-based neuroimaging

Antibodies

Antibodies used	All the antibodies used in the study are indicated in Supplementary Table S1.
Validation	All the antibodies were validated by the suppliers.

Eukaryotic cell lines

Policy information about cell lines

Cell line source(s)	All cell lines were purchased from ATCC.
Authentication	Cell lines were authenticated by ATCC.
Mycoplasma contamination	All cell lines were tested negative for Mycoplasma contamination.
Commonly misidentified lines (See ICLAC register)	No misidentified lines were used.

ChIP-seq

Data deposition

☒ Confirm that both raw and final processed data have been deposited in a public database such as GEO.

☒ Confirm that you have deposited or provided access to graph files (e.g. BED files) for the called peaks.

Data access links *May remain private before publication.*	GEO accession GSE141101 in private status and a secure token is available.
Files in database submission	2_01Q9_0058Gustave_minusAPH_FANCD2_hs-dm_i92.fastq.gz; 1_01Q8_0058Gustave_plusAPH_FANCD2_hs-dm_i87.fastq.gz; 7_01QG_0058Gustave_HC116_Pooled_Input_hs_i68.fastq.gz; FANCD2 - APH.bw; FANCD2 + APH.bw
Genome browser session (e.g. UCSC)	For data visualization, we have used the Integrative Genomics Viewer (IGV) from the Broad Institute, available at http://software.broadinstitute.org/software/igv/

Methodology

Replicates	Replicates used in the study are HCT116 - APH and HCT116 + APH.
Sequencing depth	1_01Q8_0058Gustave_plusAPH_FANCD2_hg19_i87 number of reads: 39,487,910; usable number of tags: 28,230,689<br>2_01Q9_0058Gustave_minusAPH_FANCD2_hg19_i92 number of reads: 32,270,373; usable number of tags: 23,144,212<br>Read length: 75 nt , single-end.
Antibodies	FANCD2 antibody (Novus, NB100-182)
Peak calling parameters	Only reads that pass Illumina's purity filter, align with no more than 2 mismatches, and map uniquely to the genome are used in the subsequent analysis. In addition, unless stated otherwise, duplicate reads ("PCR duplicates") are removed. Peaks were called using either the MACS or SICER algorithms. MACS default cutoff is pvalue 1e-7 for narrow peaks and 1e-1 for broad peaks, and SICER default cutoff is FDR 1e-10 with gap parameter of 600 bp. Peak filtering was performed by removing false ChIP-Seq peaks as defined within the ENCODE blacklist.
Data quality	Peaks were called using either the MACS or SICER algorithms. MACS default cutoff is pvalue 1e-7 for narrow peaks and 1e-1 for broad peaks, and SICER default cutoff is FDR 1e-10 with gap parameter of 600 bp. Peak filtering was performed by removing false ChIP-Seq peaks as defined within the ENCODE blacklist.
Software	Peaks were called using the SICER 1.1 algorithm and aligned to the human genome build hg19. Integrative Genomics Viewer (IGV) was used to visualize peaks from the genome. FANCD2 ChIP seq data were further scanned with RSAT (Regulatory Sequence Analysis Tools)-matrix-scan to identify regulatory motifs. Data have been deposited on GEO accession GSE141101.
